# Supplementary material for: Splice-Junction-Based Mapping of Alternative Isoforms in the Human Proteome
Source: Cell Rep. Author manuscript; Available in PMC 2020 Jan 15. (PMC6961840; doi:10.1016/j.celrep.2019.11.026)
Supplement: 2 [file NIHMS1546469-supplement-2.zip › DF1/PXD006675/RightAtrium/RightAtrium_21_TNS1_PHNPADILLHPTGEEDEGK.pdf]

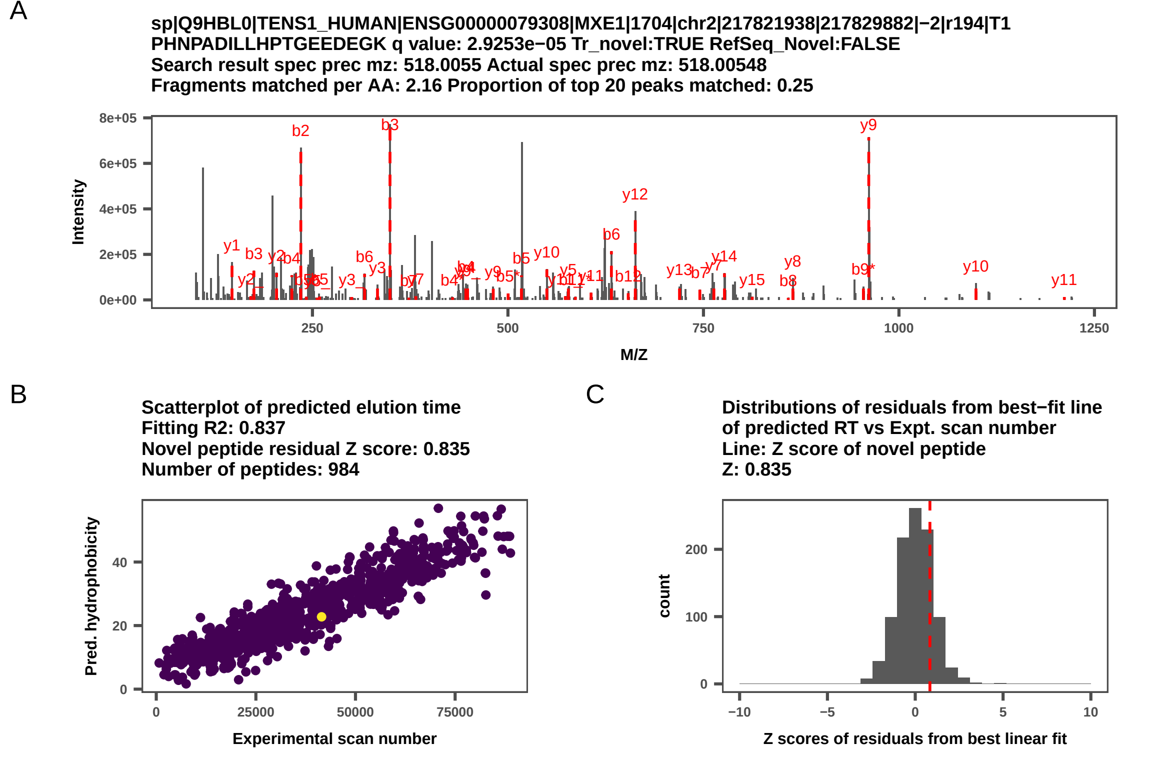
A
sp|Q9HBL0|TENS1_HUMAN|ENSG00000079308|MXE1|1704|chr2|217821938|217829882|−2|r194|T1
PHNPADILLHPTGEEDEGK q value: 2.9253e−05 Tr_novel:TRUE RefSeq_Novel:FALSE
Search result spec prec mz: 518.0055 Actual spec prec mz: 518.00548
Fragments matched per AA: 2.16 Proportion of top 20 peaks matched: 0.25
Intensity
8e+05
6e+05
4e+05
2e+05
0e+00
M/Z
250
500
750
1000
1250
b2
b3
y9
y12
b6
y10
y14
y8
b9*
y10
y11
y13
y15
b8
b12
B
Scatterplot of predicted elution time
Fitting R2: 0.837
Novel peptide residual Z score: 0.835
Number of peptides: 984
Pred. hydrophobicity
Experimental scan number
0
20
40
0
25000
50000
75000
C
Distributions of residuals from best−fit line
of predicted RT vs Expt. scan number
Line: Z score of novel peptide
Z: 0.835
count
Z scores of residuals from best linear fit
0
100
200
−10
−5
0
5
10
